# Cushing’s Syndrome Due to a Functional Thymic Neuroendocrine Tumor in Multiple Endocrine Neoplasia Type 1 Syndrome

**DOI:** 10.7759/cureus.18590

**Published:** 2021-10-07

**Authors:** Iriagbonse R Asemota, Oyintayo Ajiboye, Chineme Nwaichi, Chimezie Mbachi, Benjamin Mba

**Affiliations:** 1 Internal Medicine, John H. Stroger, Jr. Hospital of Cook County, Chicago, USA

**Keywords:** net, neuroendocrine tumor, mutiple endocrine neoplasia type, men 1, acth secreting tumor, thymic tumor, cushing syndrome, functional neuroendocrine tumor

## Abstract

Multiple endocrine neoplasia type 1 (MEN 1) syndrome is characterized by endocrinopathies and could be associated with thymic neuroendocrine tumors (NET). On rare occasions, they can be functional adrenocorticotropic hormone-secreting thymic carcinoid leading to Cushing's syndrome. In this report, we describe a case of adrenocorticotropic hormone (ACTH)-dependent Cushing’s syndrome due to a thymic NET associated with MEN type 1 syndrome. We highlight its aggressive clinical course, the premise for a high index of suspicion for an ectopic ACTH secretion, and the need for early surgical resection combined with medical therapy and alternative treatments.

## Introduction

Multiple endocrine neoplasia (MEN) 1 syndrome is a rare disorder inherited in an autosomal dominant pattern with a prevalence of 5 per 100,000 [1]. Inheritance of a mutation in the MEN 1 gene, a tumor suppressor gene mapped to chromosome 11q13 is responsible for MEN 1 syndrome and commonly predisposes individuals to tumors of the parathyroid glands, pituitary and pancreatic islet cells [2]. The clinical spectrum has now been expanded and tumors may develop in several endocrine and non-endocrine organs including foregut neuroendocrine tumors (NET) such as bronchial and thymic NET, duodenal gastrinoma, adrenal adenoma, meningioma, collagenomas, and thyroid tumors [2,3]. Thymic NET-associated tumor with MEN 1 syndrome is extremely rare, has an aggressive course, is usually nonfunctional and has a poor prognosis [3-5]. In rare cases, they may be functional and secrete adrenocorticotropic hormone (ACTH) leading to Cushing’s syndrome [4,5]. We describe a case of MEN 1 syndrome associated with thymic carcinoid with an aggressive clinical course and rapid progression to a fulminant ectopic ACTH-dependent Cushing’s syndrome.

## Case presentation

**Figure 1:**
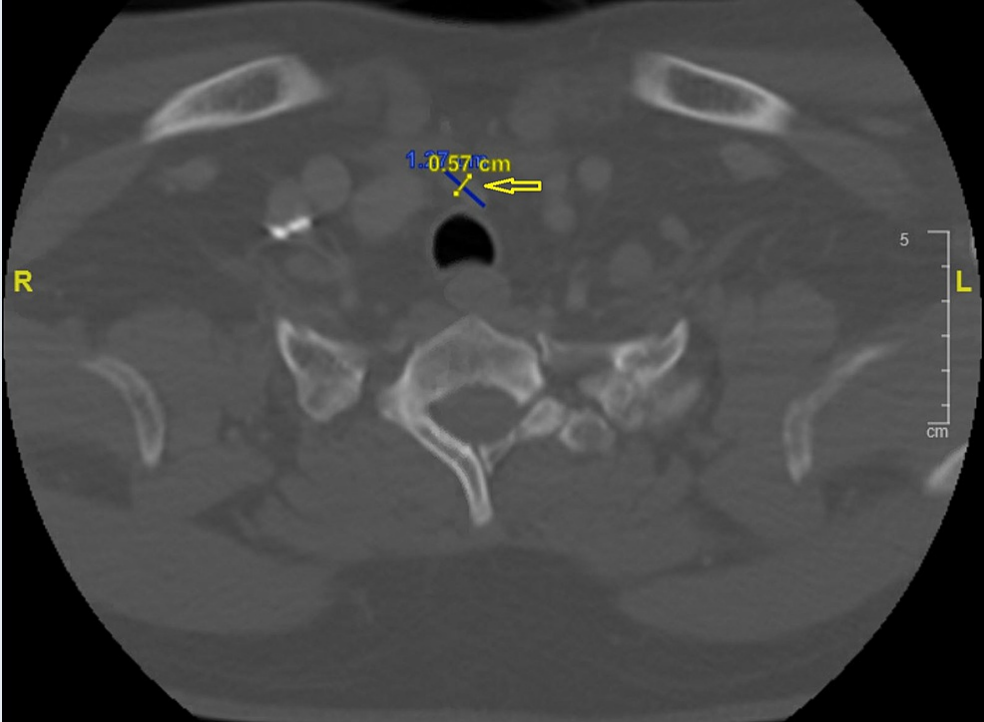
CT neck with contrast showing soft tissue nodule (yellow arrow) measuring 0.57 cm × 1.27 cm in thyroidectomy bed suggestive of tumor recurrence

**Figure 2:**
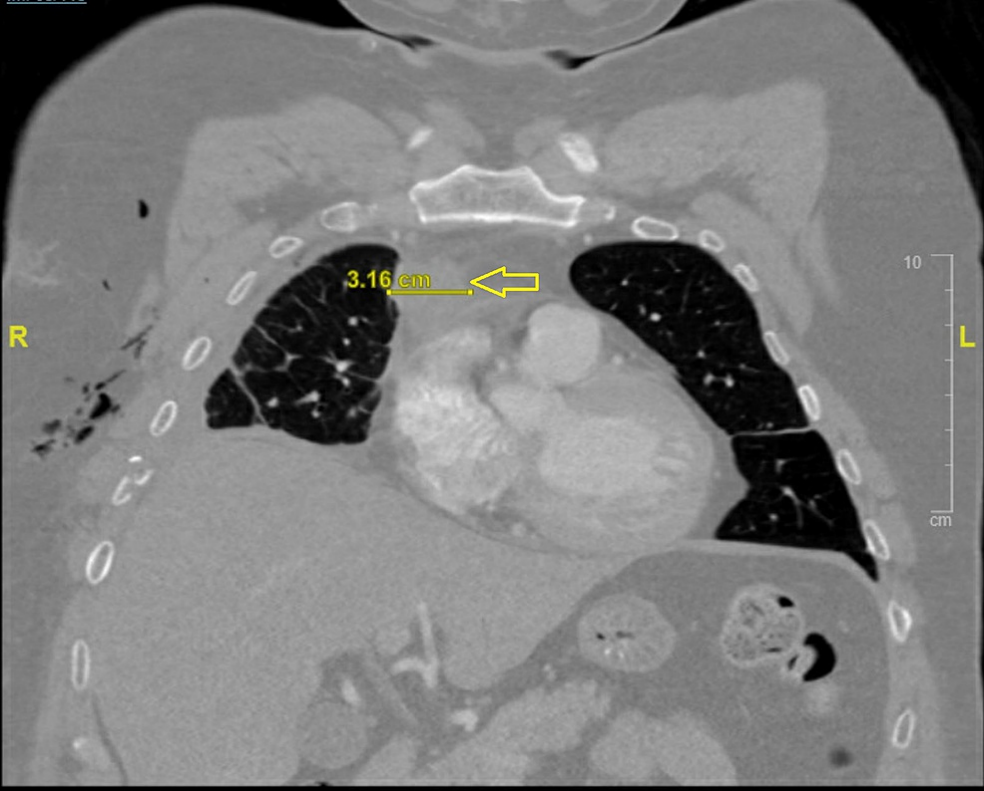
CT chest with contrast showing anterior mediastinal mass (yellow mass) measuring 3.16 cm transverse diameter

**Figure 3:**
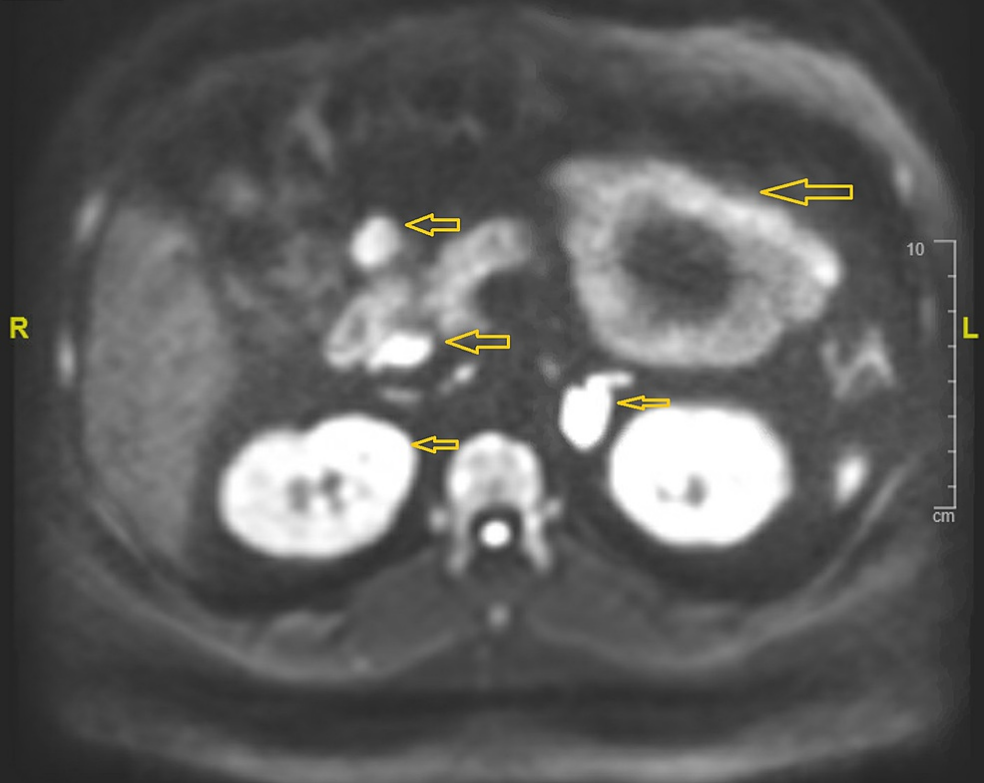
MRI abdomen with contrast showing multiple soft tissues enhancing nodules (yellow arrows) in the pancreas, duodenum, and bilateral adrenal gland

A 43-year-old Hispanic male presented to our facility with three weeks of worsening chest pain and fatigue. He had been diagnosed with papillary thyroid carcinoma, parathyroid adenoma, and bilateral nonfunctioning adrenal adenoma at a different facility, had undergone thyroidectomy and subtotal parathyroidectomy, and was discharged a week before presenting to our hospital with persistent chest pain. His vital signs were within normal limits; on physical examination, he had gynecomastia and a post-surgical anterior neck healing scar. CT of the neck and chest (Figure 1) showed a soft tissue nodule in the thyroidectomy bed suggestive of tumor recurrence. CT chest with contrast (Figure 2) also demonstrated anterior mediastinal mass suggestive of a thymic tumor. MRI abdomen with contrast (Figure 3) showed enhancing soft tissue nodules in the proximal duodenum, pancreatic uncinate process, and bilateral adrenal glands. Endocrinology workup revealed elevated prolactin, gastrin, and chromogranin as shown in Table 1.

**Table 1:** Pertinent hormonal data at initial presentation and after the onset of Cushing’s syndrome PTH: parathyroid hormone, TSH: thyroid-stimulating hormone, ACTH: adrenocorticotropic hormone.

Biochemical parameter	Normal reference	At presentation and at diagnosis of MEN 1	At diagnosis of Cushing syndrome
Prolactin (ng/ml)	2.64–13.13	239	14.4
Gastrin (pg/ml)	<100	2820.0	558.0
Insulin (µIU/ml)	1.9–23	28	-
C-peptide (ng/ml)	0.8–3.85	8.1	-
Chromogranin (ng/ml)	25–140	603.0	1753
PTH (pg/ml)	12-88	313.0	11.5
TSH (µIU/ml)	0.3–5.6	54.2	0.4
Thyroxine-T4 (ng/ml)	0.61–1.64	0.6	0.8
Cortisol free (24-hour urine)	4-50	-	8511.0
Cortisol (µg/dl) 7–9 pm	6.7–22.6	10.3	143
ACTH (pg/ml)	6-50	-	278
Aldosterone (ng/ml) 8–10 am	<28	1	<1
Renin (ng/ml)	0.25–5.2	14.3	0.58

**Figure 4:**
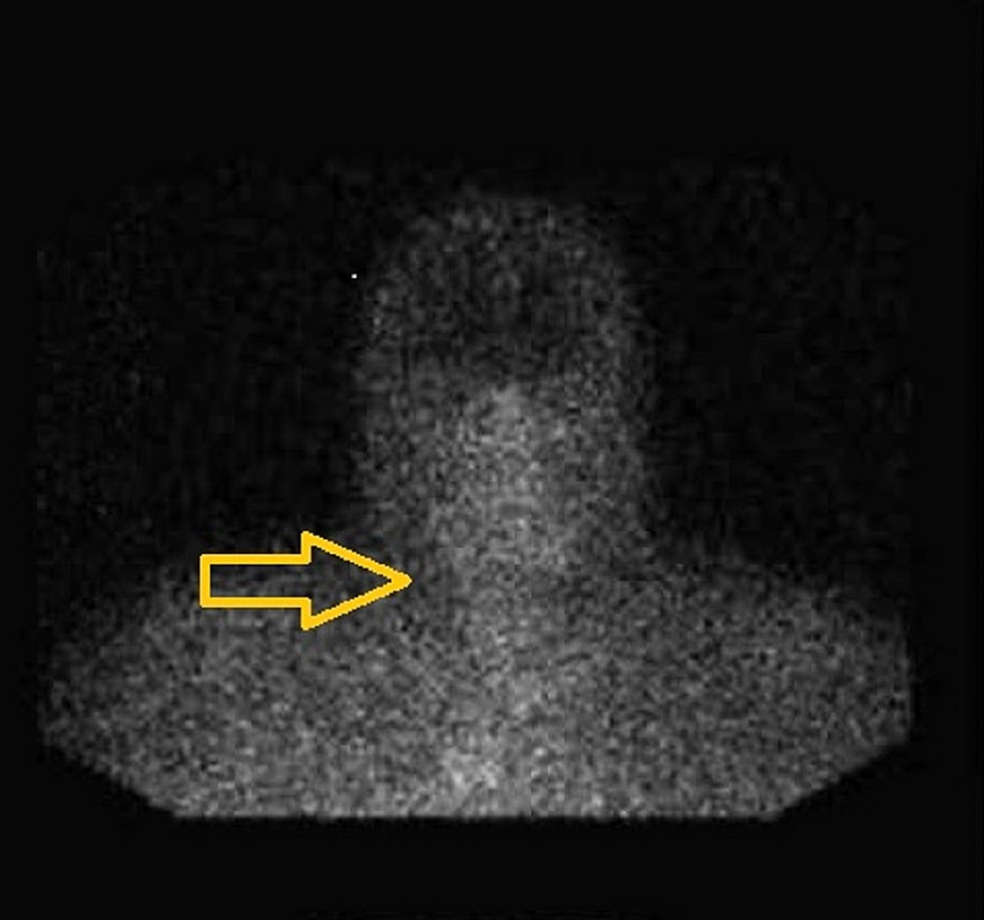
Octreoscan showing increased uptake of the indium-111 on the thymus at 24 hours

**Figure 5:**
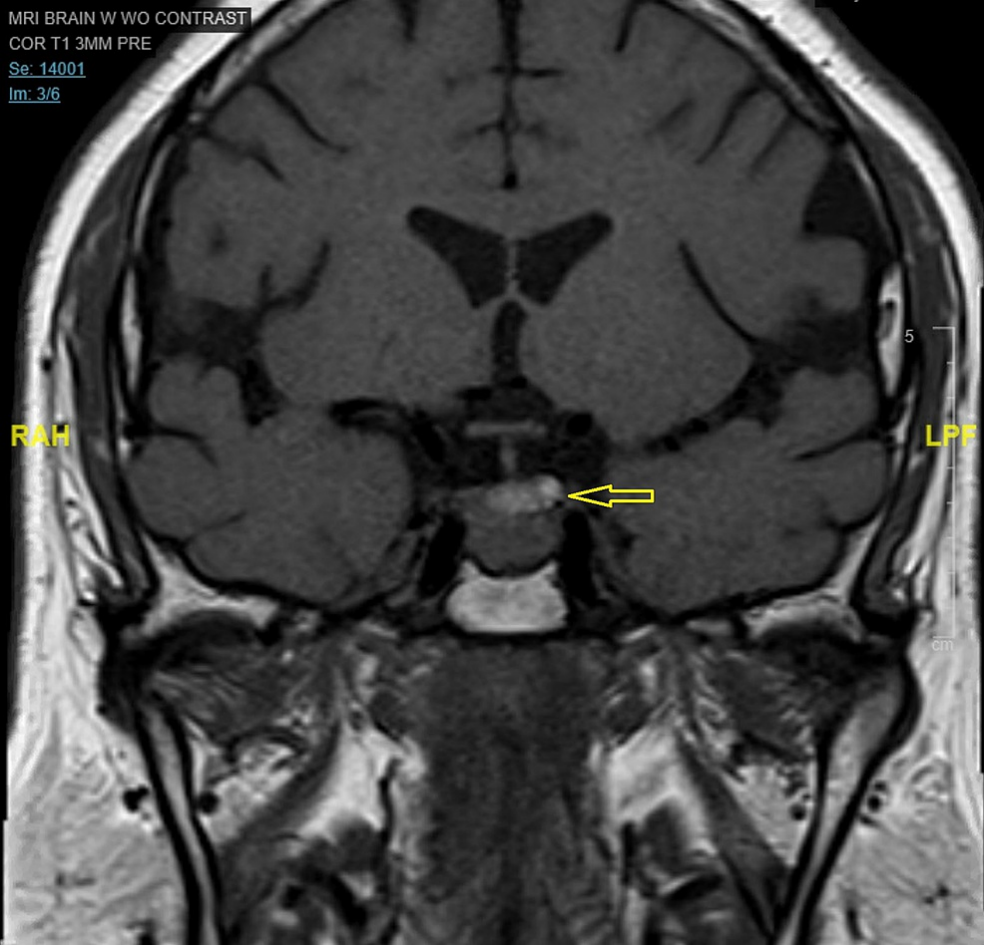
MRI of the sella-turcica revealed a hypodense lesion (yellow arrow) in the inferior pole of the Sella without a supra-sellar extension

An octreotide scan showed increased uptake in the thymus suggestive of a NET (Figure 4) and an MRI of the sella-turcica (Figure 5) revealed a hypodense lesion in the inferior pole of the Sella without a supra-sella extension. A provisional diagnosis of MEN type 1 syndrome was made and consisted of hyperparathyroidism, a prolactin-producing pituitary adenoma, a pancreatic gastrinoma, and a thymic mass suggestive of thymic NET. He had total parathyroidectomy plus autotransplantation and resection of residual thyroid nodules. Then started treatment with cabergoline, pantoprazole, levothyroxine, calcitriol, ergocalciferol, and calcium carbonate. Although he was planned for thymic mass resection at the same surgery, it had to be rescheduled due to the unexpected length and duration of the surgery.

He was readmitted two weeks later for resection of the anterior mediastinal mass via right video-assisted thoracoscopic surgery (VATS). Intraoperatively, there was an unresectable bulky immobile anterior mediastinal mass invading the underlying confluence of the innominate veins as they form the superior vena cava (SVC) with extensive adhesions between the lung, chest wall, and mediastinum. A tissue biopsy was obtained which later revealed an invasive poorly differentiated neuroendocrine tumor (small cell carcinoma) with Ki 67 >50% and the cells immunostained for chromogranin and synaptophysin. Subsequent genetic testing using the cancer next-expanded 2018 panel was positive for a mutation in the MEN-1 gene. He was started on chemotherapy and received the first cycle of carboplatin and etoposide with a plan for additional chemotherapy and radiotherapy. Unfortunately, not adhering to the treatment course, he was readmitted two months later with severe dyspnea, generalized edema, and pronounced abdominal striae. On evaluation, he had elevated 24-hour urine cortisol, with no significant decrease in cortisol level on low and high dose dexamethasone suppression test. Further workup revealed elevated ACTH (as shown in Table 1) and subsequent immunostaining of the previously biopsied thymic mass was positive for ACTH.

It became apparent that he had Cushing's syndrome due to an ACTH producing thymic carcinoid and was treated with spironolactone and ketoconazole. He received the second cycle of carboplatin and etoposide on the 12th day on admission, however, on the 16th day on admission he developed acute psychosis due to Cushing’s syndrome requiring admission to the intensive care unit. His clinical course was complicated by septic shock due to extended-spectrum beta-lactamase (ESBL) *Escherichia coli* and *Enterobacter cloacae* with acute respiratory distress syndrome requiring intubation. Later passed away on the 21st day on admission following a cardiac arrest. 

## Discussion

MEN 1 syndrome typically presents with primary hyperparathyroidism in 95% of cases, pancreatic islet cell tumor in 40%, and anterior pituitary adenoma in 30% [1]. About 25% of thymic NET is associated with MEN 1 syndrome, while only 3-8% of patients with MEN 1 syndrome develop thymic NET. As a result, the presence of thymic NET at initial presentation like in this patient is rare [3,4]. A functioning thymic NET in MEN 1 syndrome occurring as ectopic ACTH-dependent Cushing’s syndrome is even rarer and may be diagnosed during the disease course or at initial presentation [3].

According to 2015 WHO classification for thymic tumors, NET arising from the thymus can be categorized as typical carcinoids (low grade), atypical carcinoids (intermediate grade), large cell neuroendocrine carcinoma (high grade), and small cell carcinoma (high grade). The low and intermediate-grade are referred to as well-differentiated while the high-grade tumor is poorly differentiated [4]. These tumors have an aggressive clinical behavior and a poor overall survival rate with a 10-year survival of about 10-35% [6]. Studies have reported higher mortality in MEN 1 syndrome when associated with thymic NET compared to those with bronchial NET [4], hence, a need for a more aggressive approach in these patients. It is now recommended for patients with MEN 1 syndrome to have CT or MRI of the chest every one to two years for detection of thymic and bronchopulmonary NET [2]. Though rare for thymic NET to secrete ACTH especially in the setting of a rare disease entity such as MEN 1 syndrome, a clinical consideration and high index of suspicion should be entertained especially when Cushing’s syndrome is suspected and can be confirmed with an elevated ACTH level and immunohistochemistry staining positive for ACTH as seen in this patient [7].

Local tumor invasion of mediastinal structures and local recurrence are characteristic of thymic neuroendocrine carcinoma [7]. Therefore, early surgical resection with medical hormonal therapy when indicated is essential in order to forestall post-surgical adrenal insufficiency and control other associated endocrinopathies [7]. Complete surgical resection is desirable in order to achieve symptoms resolution and prevent recurrence [2,6]; however, if the tumor is unresectable, due to the involvement of delicate surrounding vasculature and/or airway structures as seen in our patient, chemotherapy and radiotherapy are recommended treatment modalities to ameliorate disease progression and distant metastasis [2]. For patients with poorly differentiated thymic NET like in our patient, platinum/etoposide-based therapy is a recommended option [4]. There have only been few cases of patients with advanced disease who received neoadjuvant or salvage therapy, while this may be helpful to improve survival, very few treatment options are available and their role in improving overall survival is not clear [4]. In addition, the available treatment modalities are not based on clear evidence due to a lack of prospective clinical trials given the rarity of thymic NET [4], and of note, the treatment of thymic NET in patients with MEN 1 is similar to that in non-MEN 1 patients [2].

## Conclusions

Thymic carcinoid tumors in MEN 1 syndrome have an aggressive course especially as a functional ACTH secreting neuroendocrine tumor. In patients who have Cushing’s syndrome and a thymic carcinoid, there should be a high index of suspicion for an ectopic ACTH production and immunostaining of thymic tissue should be done to confirm the diagnosis. In addition, timely neoadjuvant chemotherapy and radiation therapy may be helpful in advanced cases with the possibility to improve prognosis.
